# Integrated transcriptome and metabolome analysis reveals the molecular mechanisms of drought response in *Setaria italica* during the booting stage

**DOI:** 10.1186/s12870-026-08353-9

**Published:** 2026-02-26

**Authors:** Meihong Huang, Jiujun Du, Yu Zhao, Jihan Cui, Min Zhang, Fengyan Cao, Jingxin Wang, Cheng Chu, Shunguo Li, Xueyan Xia

**Affiliations:** Institute of Millet Crops, Hebei Academy of Agriculture and Forestry Sciences/Key Laboratory of Genetic Improvement and Utilization for Featured Coarse Cereals (Co-Construction by Ministry and Province), Ministry of Agriculture and Rural Affairs/The Key Research Laboratory of Minor Cereal Crops of Hebei Province, Shijiazhuang, 050035 China

**Keywords:** *Setariaitalica*, Drought, Photosynthetic parameters, Transcription-metabolism analysis

## Abstract

**Background:**

Drought is one of the most severe and prevalent abiotic stresses affecting crop production. Foxtail millet (*Setaria italica*), an important cereal crop in the *Poaceae* family, is considered an ideal crop for future sustainable agriculture due to its remarkable drought tolerance, water-use efficiency (WUE), and strong adaptability to poor soils. The booting stage is widely acknowledged as a pivotal developmental phase in foxtail millet, and this consensus has been well established in existing research. However, the molecular mechanisms underlying the drought response of foxtail millet during this critical stage remain poorly elucidated. In the present study, we employed an integrated approach combining transcriptomic and metabolomic sequencing to identify the core regulatory mechanisms and key genes governing the drought stress response in foxtail millet at the booting stage.

**Results:**

In this study, phenotypic variations, gene expression profiles, and metabolite accumulation patterns in foxtail millet were determined following drought treatments at different growth stages. Among these stages, drought stress imposed at the booting stage resulted in the most severe yield reduction of 34.94%, accompanied by the identification of 566 differentially expressed genes (DEGs) — the highest number across all tested stages. These findings indicated that the booting stage was the most vulnerable period to drought stress during foxtail millet growth. Furthermore, analyses of photosynthetic parameters and yield-related traits revealed that drought stress at the booting stage significantly inhibited photosynthetic capacity, which may represent a critical factor contributing to the yield decline observed in foxtail millet subjected to drought stress during the booting stage. Multi-omics integration analysis identified three core responsive pathways: phenylpropanoid biosynthesis, photosynthetic carbon fixation, and glyoxylate and dicarboxylate metabolism. Based on these findings, we constructed a drought resistance regulatory network model for the booting stage, revealing significant expression changes *GAPA*, *ALDO*, *tktA*, *glpx*, *PRK*, *RPE*, *rbcS*, *maeB*, *GLUL*, and *gcvT*. At the metabolic level, we observed significant accumulation of metabolites such as sinapyl alcohol, aconitic acid, citric acid, isocitric acid, and mesaconic acid, while the contents of methyl isoeugenol, eugenol, *p*-coumaric acid, chlorogenic acid, sedoheptulose, aspartic acid, and malic acid were markedly reduced. Most notably, this study revealed that downregulation of *gcvT* expression may restrict ammonia (NH_3_) supply, thereby activating the methanesulfonate synthesis pathway as an alternative nitrogen (N) source. This discovery provided novel insights into N metabolic remodeling under drought stress in plants.

**Conclusions:**

This study laid the foundation for preliminary exploration of the molecular mechanisms underlying drought stress response during the booting stage in foxtail millet. Furthermore, these findings had identified valuable key candidate genes that may contribute to the breeding of high-yield and drought-resistant foxtail millet varieties.

**Supplementary Information:**

The online version contains supplementary material available at 10.1186/s12870-026-08353-9.

## Introduction

Drought, salinity, heavy metals, and other abiotic stress factors cause significant yield and quality losses in plants [1, 2], among them, drought is one of the most common and severe abiotic stress factors, which can lead to damage to the plant photosynthetic system and imbalance in dry matter distribution, ultimately resulting in significant yield reduction [3, 4]. Furthermore, drought weakens plant stress resistance, increases the risk of pests and diseases, and affects grain quality [5, 6]. Its adverse effects severely constrain plant growth and crop productivity in arid and semi-arid regions [7]. Despite the complexity of drought tolerance, significant progress has been made in understanding the drought adaptation mechanisms of plants [8, 9]. Plant drought tolerance is primarily regulated through changes in plant morphology and growth, an adaptation process involving complex gene expression regulation and associated molecular pathways [10, 11]. Drought stress impairs photosynthesis by reducing both leaf area per unit and photosynthetic rate [12, 13]. The decrease in photosynthetic rate occurs mainly through stomatal closure or metabolic damage. Under drought stress, when intracellular CO_2_ concentration becomes limited, the ongoing light reactions of photosynthesis cause an accumulation of reduced components of the photosynthetic electron transport chain, which have the potential to reduce molecular oxygen, leading to the production of reactive oxygen species (ROS) [14–17].

Foxtail millet (*Setaria italica*), originating in China, is an important economic crop within the grass family with a long history of cultivation [17]. As one of the cereals with the highest water use efficiency (WUE) [18], foxtail millet exhibits remarkable drought tolerance, making it an ideal system for studying plant drought resistance and stress response mechanisms. Characterized by its short growth cycle, strong drought tolerance, and tolerance to poor soils [19], it is one of the most important minor cereal crops in the arid and semi-arid regions of northern China. It plays a crucial role in rainfed agroecosystems, agricultural production diversity, and food security [20]. However, the drought resistance of foxtail millet exhibits significant stage-specific expression. This trait is particularly pronounced during water-sensitive developmental stages such as the booting phase, where drought stress imposes substantial impacts on growth and developmental processes [21]. Therefore, enhancing crop WUE through genetic approaches is a major focus of current breeding research. This necessitates a systematic elucidation of the molecular mechanisms underlying foxtail millet’s response to drought stress.

To gain deeper insights into the dynamic relationships between gene expression and metabolite synthesis, multi-omics integrated analyses have been conducted in various crops such as *Lolium perenne* [22], *Oryza sativa* [23], *Zea mays* [24], *Solanum tuberosum* [25], and *Gossypium* [26] to uncover their drought resistance mechanisms. Foxtail millet has emerged as an ideal model crop for investigating the molecular mechanisms underlying drought resistance [27]. However, research in this area remains relatively limited for foxtail millet. In existing studies, extensive transcriptome sequencing analyses have been conducted on foxtail millet, leading to the identification of both protein-coding and non-coding RNAs involved in the drought response [29]. These studies have revealed that the expression levels of genes associated with the phenylalanine, glutathione, and light-responsive pathways are altered in foxtail millet under drought stress [28, 29]. Meanwhile, enhanced lignin metabolism, promotion of fatty acid conversion into cutin and wax, and improvement of the ascorbate cycle have been recognized as key strategies underlying drought adaptation in foxtail millet [30]. In addition, researchers have further investigated gene expression patterns and metabolite accumulation profiles in drought-stressed foxtail millet by employing an integrated transcriptomic and metabolomic analysis approach [31]. To date, there are no comprehensive studies that characterize the integrated transcriptomic and metabolomic responses specifically at the booting stage under drought in foxtail millet.

This study used *Jigu 45* as experimental material. Through systematic measurements of photosynthetic characteristics and yield parameters, we identified for the first time that the booting stage is the critical period determining photosynthetic product accumulation and yield formation in millet. Based on integrated multi-omics analysis, we comprehensively elucidated the core metabolic pathways involved in foxtail millet’s response to drought stress during the booting stage and identified a series of key differentially expressed genes (DEGs) and differentially accumulated metabolites (DAMs). These findings not only provided new theoretical insights into the physiological mechanisms of drought resistance in foxtail millet but also offered important molecular targets and practical guidance for breeding drought-resistant varieties and optimizing cultivation techniques. The innovative discoveries of this study hold significant scientific implications and practical value for enhancing drought tolerance in foxtail millet and ensuring stable grain production in arid regions.

## Result

### Drought inhibits photosynthetic and yield-related parameters in millet

**Fig. 1:**
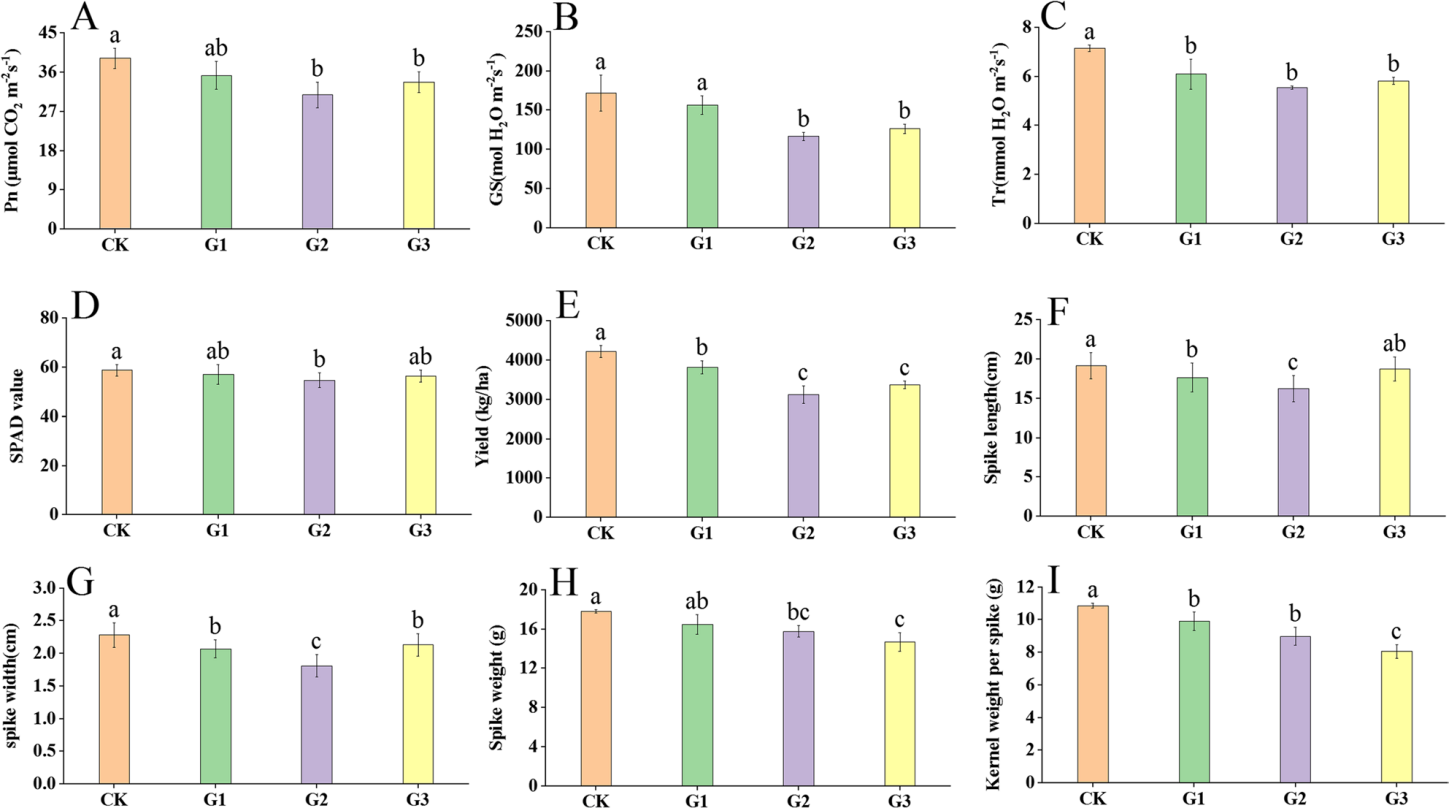
Phenotypic characteristics, photosynthetic traits, and yield-related indices of foxtail millet under drought stress. CK: Normal irrigation; G1: Drought at jointing stage; G2: Drought at booting stage; G3: Drought at filling stage. **A** to **I** represent net photosynthetic rate (Pn), conductance to H_2_O (Gs), transpiration rate (Tr), SPAD value, yield, spike length, spike width, spike weight, and kernel weight per spike, respectively. ANOVA was used to analyze the statistical significance of data comparisons and different letters in the same figure indicate significant differences among treatments (Mean ± SE, *p*-value < 0.05)

The photosynthetic indices of foxtail millet showed inhibition after drought treatment, with photosynthetic rate, conductance to H_2_O, transpiration rate, SPAD value decreasing by a maximum of 21.5% (G2), 32.11% (G2), 22.37% (G2), and 6.88% (G2), respectively (Fig. 1A ~ D). The yield indices of foxtail millet showed inhibition after drought treatment, with yield, spike length, spike diameter, spike weight, and kernel weight per spike decreasing by a maximum of 25.9% (G2), 15.23% (G2), 20.61% (G2), 26.0% (G2), 17.60% (G2) respectively (Fig. 1E ~ I). The results demonstrated that drought stress during the booting stage exerts the most pronounced adverse effects on photosynthetic and yield-related parameters in foxtail millet.

### Transcriptome analysis

**Fig. 2:**
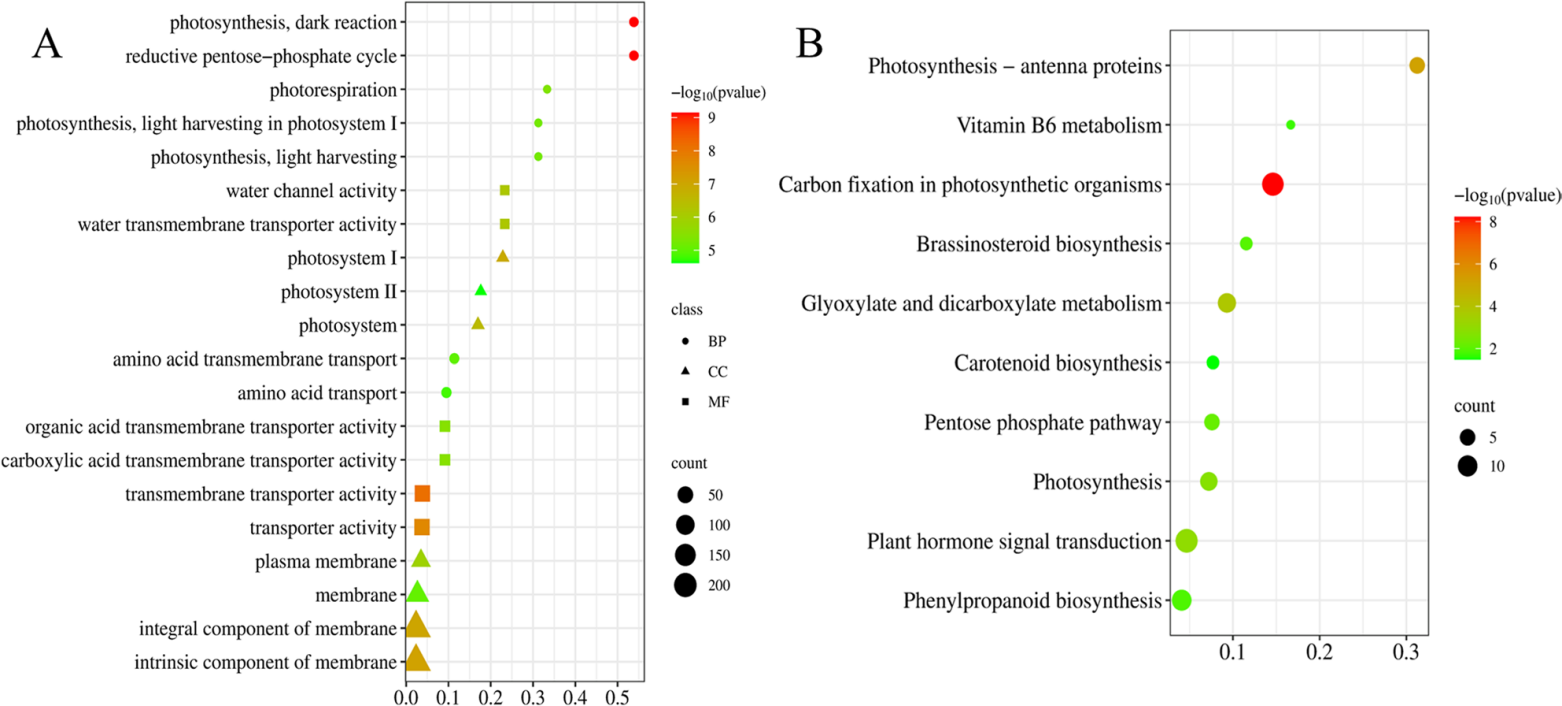
GO and KEGG enrichment analysis of the DEGs between the G2 vs. CK group. **A** GO enrichment analysis result chart. BP: Biological process; CC: Cellular component; MF: Molecular function. **B** KEGG enrichment analysis result chart. The horizontal axis represents the rich factor of DEGs, and the vertical axis represents the name of the GO term or KEGG pathway. The size of the dot represents the number of DEGs, and the color of the dot represents the enrichment significance. Terms and pathways were filtered with a threshold of *p*-value < 0.05. Dot size denotes the number of enriched genes, while color corresponds to -log₁₀(*p*-value)

The number of clean reads ranged from 43,361,614 to 55,642,796, with Q30 values exceeding 94% (sequencing error rate < 0.028%). RNA-seq analysis revealed the substantial changes of transcription level of foxtail millet under drought stress in different periods. The number of DEGs was significantly higher under drought stress at the booting stage (G2), with 221 up- and 345 downregulated genes, compared to the normal irrigation group. In contrast, 268 (with 147 up- and 121 downregulated) and two DEGs (two upregulated) were identified in the G1 and G3 treatment groups, respectively. The extremely low number of DEGs in the G3 group might be attributed to the relatively late timing of the drought treatment application (Supplementary table S1). This was also consistent with the results of index determination after drought treatment, suggesting that the booting stage may be the most critical for foxtail millet. The Gene Ontology (GO) enrichment analysis showed the identified DEGs of the G2 vs. CK group were significantly enriched in 31 biological processes of GO terms. Many of which were associated with photosynthesis. The most prominent categories include component of membrane (220 DEGs), integral component of membrane (218 DEGs), and membrane (104 DEGs) (Fig. 2A, Supplementary table S2). The Kyoto Encyclopedia of Genes and Genomes (KEGG) pathway analysis highlighted 10 significantly enriched metabolic pathways, including plant hormone signal transduction, carbon fixation in photosynthetic organisms, and phenylpropanoid biosynthesis (Fig. 2B, Supplementary table S3). For the G1 vs. CK group, GO enrichment analysis revealed that DEGs were primarily associated with biological processes including substance transport, metabolic regulation, and stress response, among which substance transport and metabolic regulation represented the two most prominent functional categories. Specifically, substance transport processes encompassed the transmembrane transport and transport of amino acids, organic acids, carboxylic acids, and other key metabolites, while metabolic regulation involved the metabolism of spermine, polyamines, glutathione, and various other bioactive substances. Notably, four stress resistance-related GO terms were identified, namely response to temperature stimulus, response to cold, cellular response to heat, and response to abiotic stimulus, which are closely linked to plant adaptation to adverse environments. However, no significantly enriched pathways were detected in the KEGG pathway analysis for this group. In contrast, due to the extremely limited number of DEGs in the G3 vs. CK group, only three GO terms were enriched, including apoplast, extracellular region, and manganese ion binding, with no significantly enriched KEGG pathways observed either.

### Metabolomic profiling of foxtail millet responses to drought stress at the booting stage

**Fig. 3:**
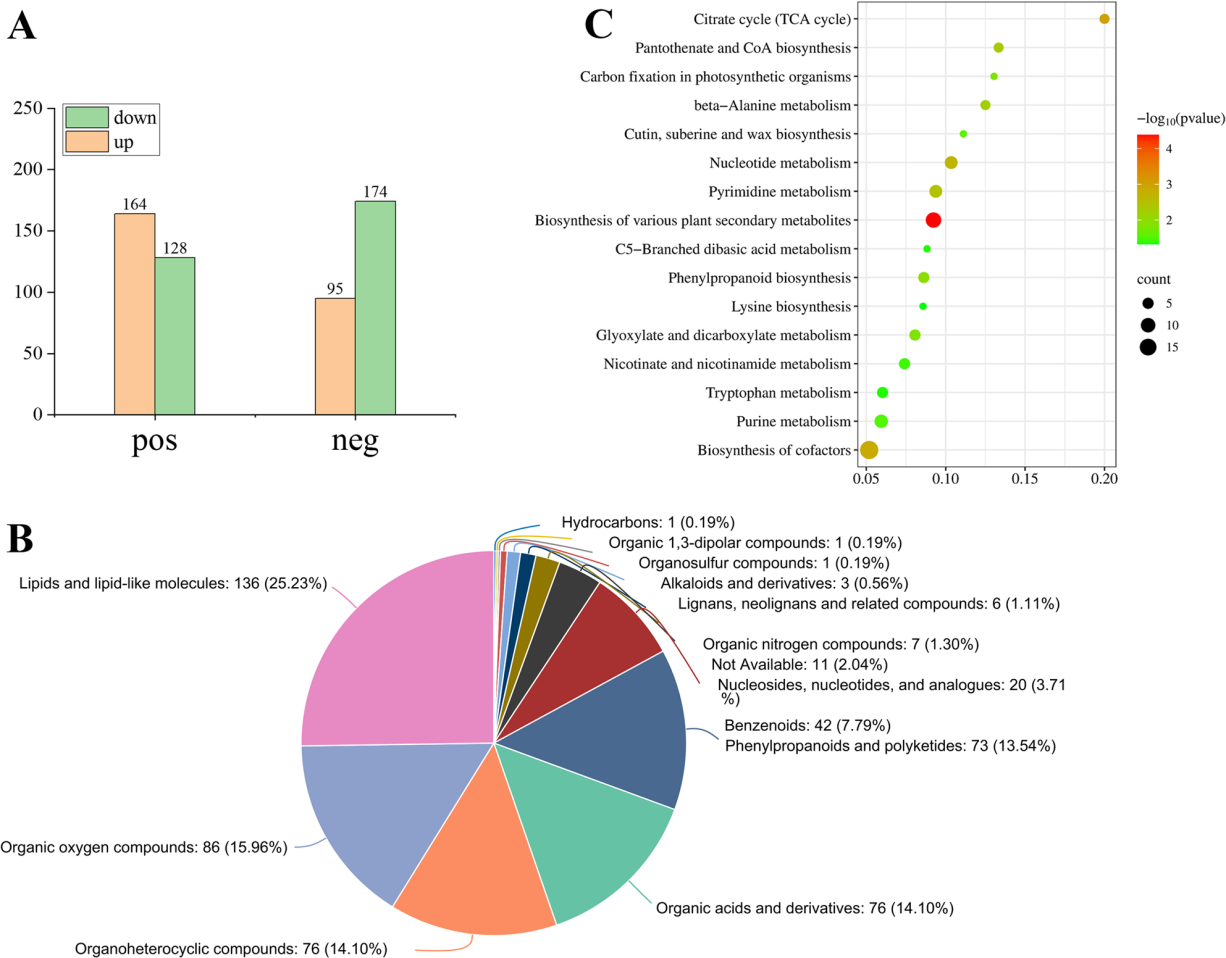
Metabolomics data profiles. **A** Metabolite difference statistics chart of the G2 vs. CK group. **B** The classification of annotated metabolites in the G2 vs. CK group. **C** KEGG enrichment analysis of the DAMs between the G2 vs. CK group. The horizontal axis represents the rich factor of DAMs, and the vertical axis represents the pathway. The size of the dot represents the number of DAMs, and the color of the dot represents the enrichment significance

To investigate the changes in metabolites during the booting stage, a widely-targeted metabolomics approach was employed for metabolite profiling. Orthogonal partial least squares discriminant analysis (OPLS-DA) revealed R^2^Y = 0.999 and Q^2^ > 0.97 in all comparisons, confirming the high accuracy of the model. A total of 2,837 metabolites were detected, among which 561 were DAMs, including 292 cations and 269 anions (VIP ≥ 1, *p* < 0.05, and |log_2_FC| ≥ 1). Among the cations, 164 DAMs were upregulated and 128 were downregulated. Among the anions, 95 DAMs were upregulated and 174 were downregulated (Fig. 3A). All 561 DAMs obtained from identification were assigned to the HMDB database, which were matched and classified into 11 HMDB super classes. Of these, which contained lipids and lipid-like molecules (136), organic oxygen compounds (86), organic acids and derivatives (76), and organic heterocyclic compounds (76) (Fig. 3B).

DAMs identified between the different groups were subjected to KEGG enrichment analysis (Fig. 3C, Supplementary table S4). The main metabolic pathways of the DAMs in the G2 and CK groups were associated with the citrate cycle (TCA cycle), pantothenate and CoA biosynthesis, and carbon fixation in photosynthetic organisms. Moreover, carbon fixation in photosynthetic organisms, glyoxylate and dicarboxylate metabolism, and phenylpropanoid biosynthesis were observed in the DAMs between metabolomic and transcriptome.

### Key pathways revealed by multi-omics integration

**Fig. 4:**
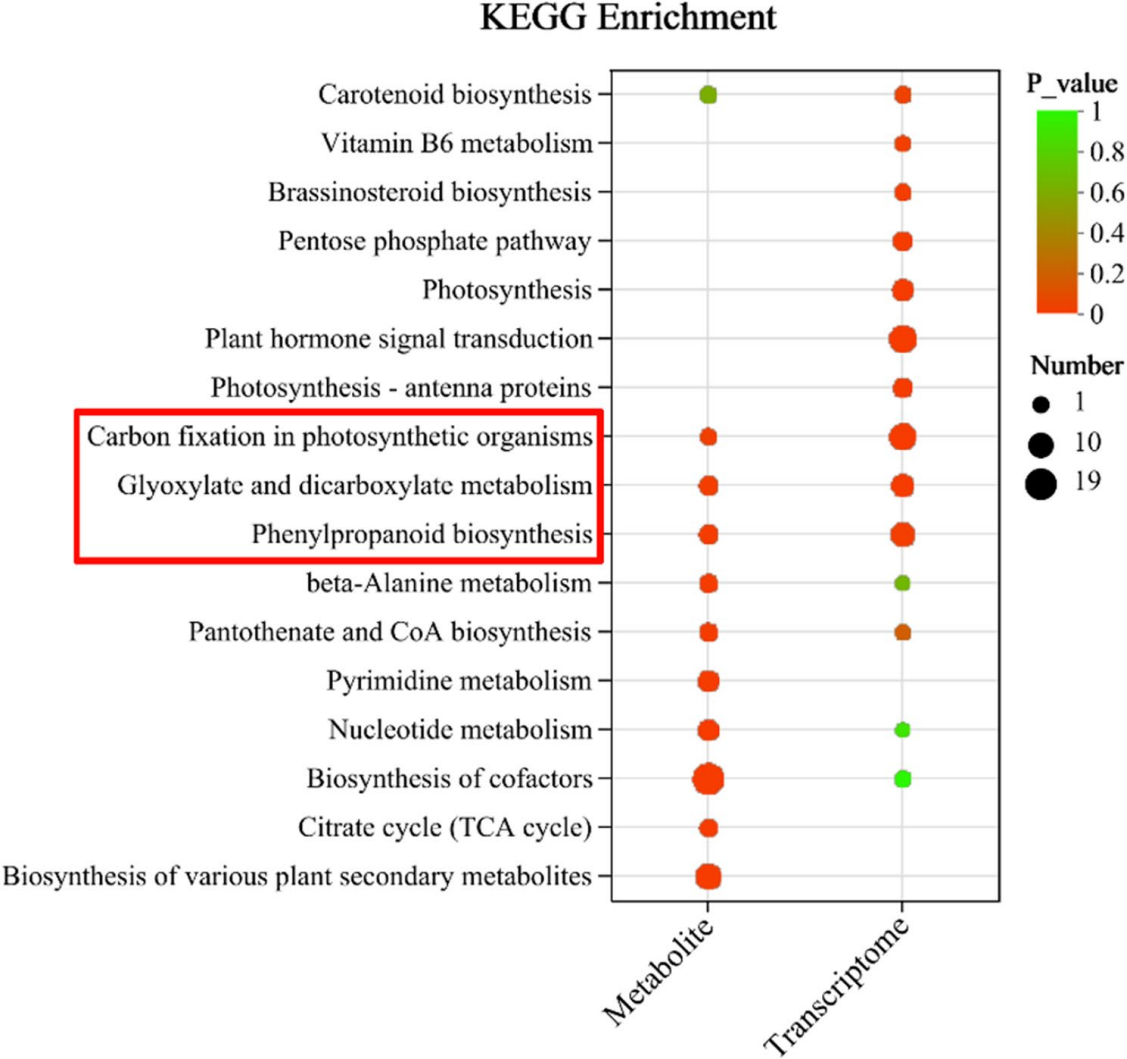
Metabolic-transcriptional integrated KEGG enrichment plot. The red boxes represent the intersection of metabolic and transcriptional pathways

A combined analysis was conducted based on DEGs and DAMs. The number of common KEGG pathways for both DEGs and DAMs in the comparison groups of G2 vs. CK were three, which included phenylpropanoid biosynthesis, carbon fixation in photosynthetic organisms, and glyoxylate and dicarboxylate metabolism (Fig. 4). Based on the integrated analysis of metabolomic and transcriptomic data, we concluded that these three pathways are the most significantly affected core pathways in foxtail millet in response to drought stress at the booting stage.

**Fig. 5:**
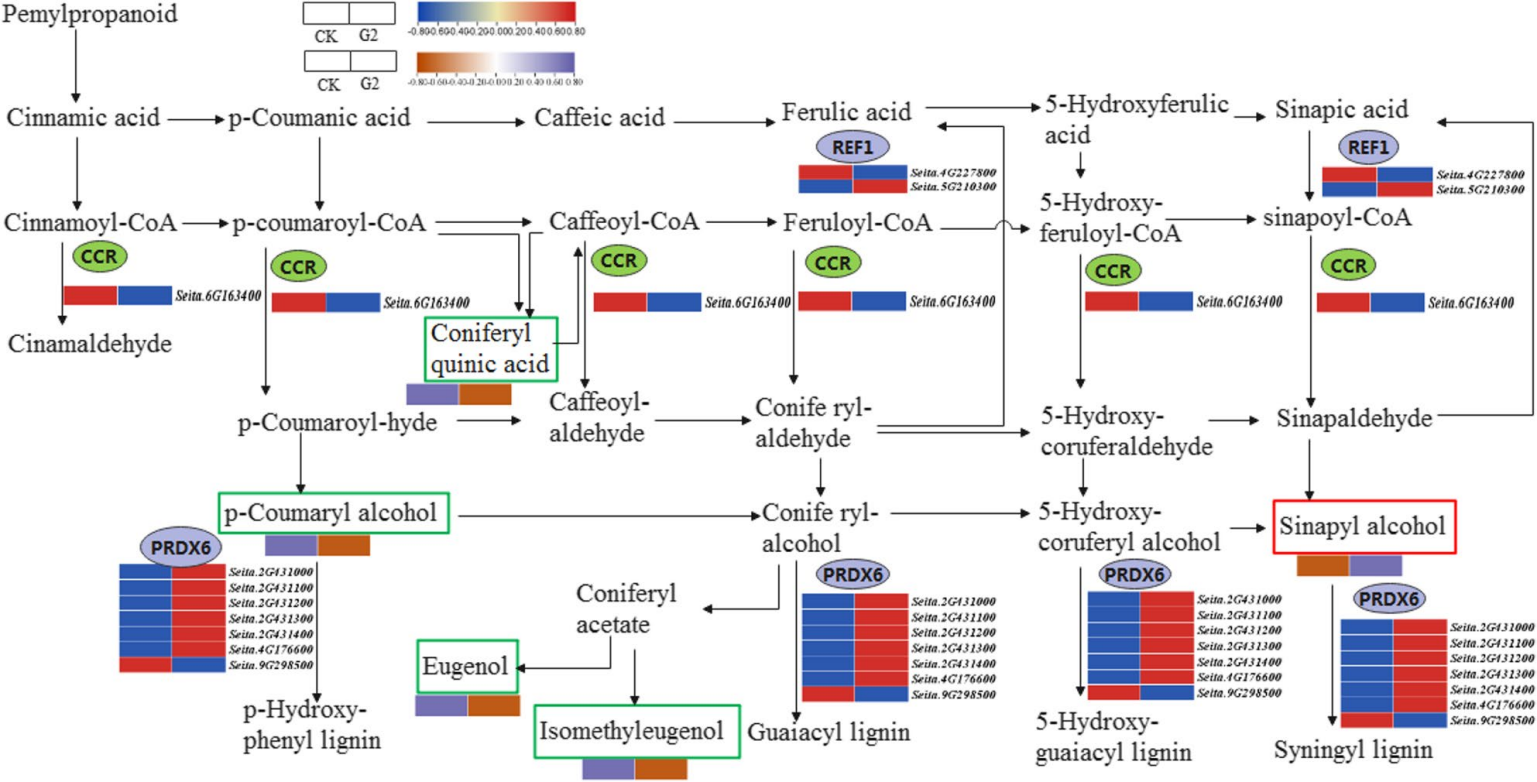
Changes in phenylpropanoid biosynthesis pathway genes and metabolites under drought stress during the booting stage. The blue and red color blocks represent the fragments per kilobase of transcript per million mapped reads (FPKM) values of DEGs, while the brownish red and purple color blocks represent changes in metabolite content

In the phenylpropanoid biosynthesis pathway, ten DEGs and five DAMs were differentially expressed or accumulated, respectively. Among them, the cinnamoyl-coenzyme a reductase (*CCR*) (Seita.6G163400), which controls cinnamaldehyde synthesis, was downregulated. The peroxidase (*PRDX6*) (Seita.2G431000, Seita.2G431100, Seita.2G431200, Seita.2G431300, Seita.2G431400, and Seita.4G176600 upregulated, Seita.9G298500 downregulated) was upregulated bidirectionally. Additionally, both *CCR* and *PRDX6* genes simultaneously regulate the synthesis of p-Hydroxy-phenyl lignin, eugenol, and guaiacyl lignin. Current studies have shown that impaired plant cell development reduces drought tolerance [32]. The coniferyl-aldehyde dehydrogenase (*REF1*) (Seita.4G227800) showed a downregulation trend, while Seita.5G210300 was upregulated. The five DAMs were eugenol, *p*-coumaric acid, chlorogenic acid, sinapyl alcohol, and methyl isoeugenol. Among them, sinapyl alcohol was upregulated, while the other metabolites were downregulated in their accumulation. This also indicated that different types of lignin exhibit distinct variation trends in their contents (Fig. 5).

**Fig. 6:**
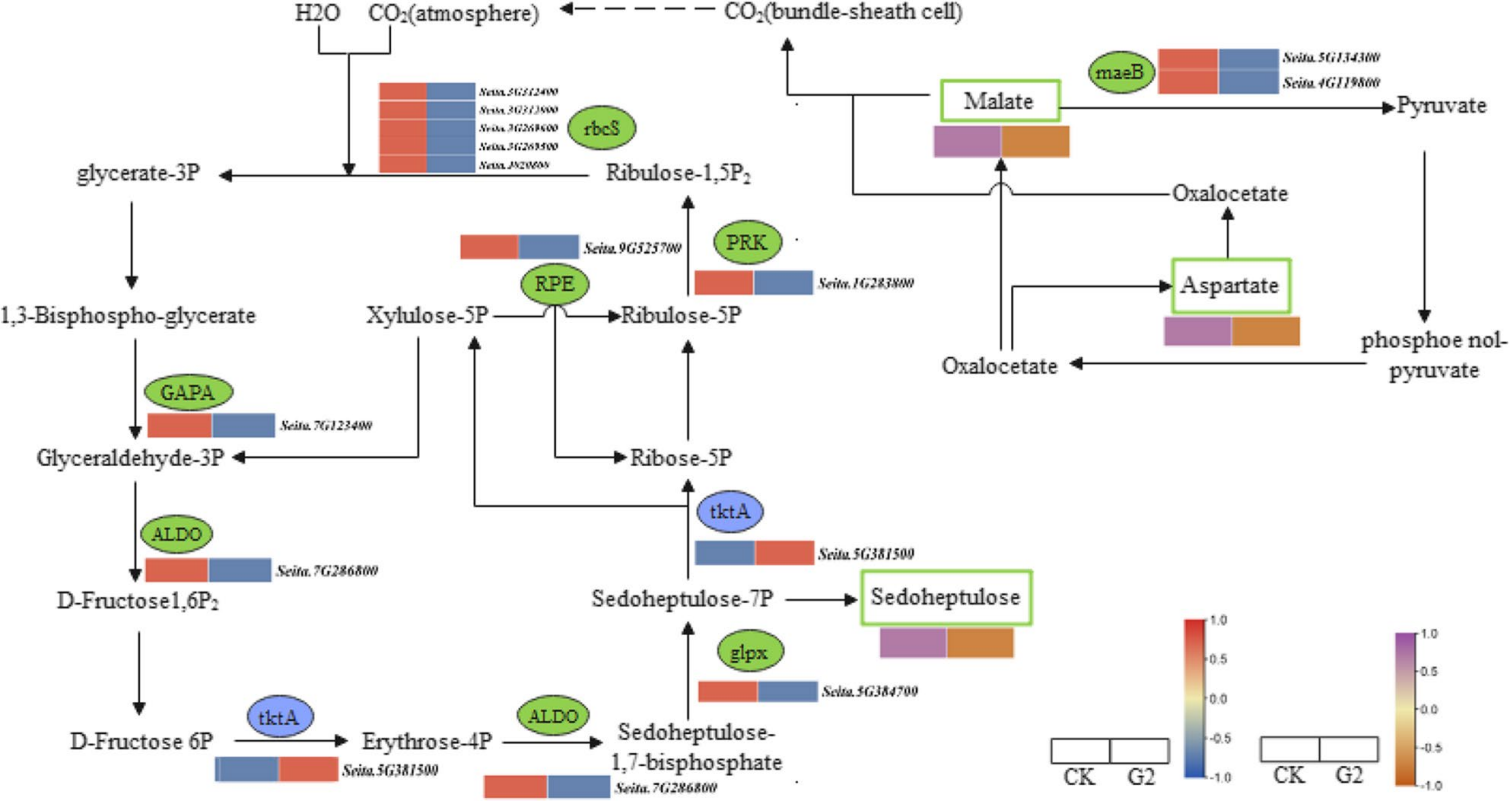
Changes in carbon fixation in photosynthetic organisms pathway genes and metabolites under drought stress during the booting stage. The blue and red color blocks represent the FPKM values of DEGs, while the brownish red and purple color blocks represent changes in metabolite content

In the carbon fixation pathway of photosynthetic organisms, thirteen DEGs and three metabolites were differentially expressed or accumulated, respectively (Fig. 6). Among them, the transketolase (*tkta*) (Seita.5G381500) showed an upregulation trend; while the glyceraldehyde-3-phosphate dehydrogenase (NADP+) (*GAPA*) (Seita.7G123400), fructose-bisphosphate aldolase (*ALDO*) (Seita.7G286800), fructose-sedoheptulose-1,7-bisphosphatase (*glpx*) (Seita.5G384700), ribulose-phosphate 3-epimerase (*RPE*) (Seita.9G525700), phosphoribulokinase (*PRK*) (Seita.1G283800), ribulose-diphosphate carboxylase (*rbcS*) (Seita.3G312000, Seita.3G312400, Seita.3G269600, Seita.J020800 and Seita.3G269500), as well as malate dehydrogenase (*maeB*) (Seita.5G134300 and Seita.4G119800) showed a downregulation trend. The three DAMs were sedoheptulose, aspartate, and malic acid, all of which showed a downregulation trend in their accumulation.

**Fig. 7:**
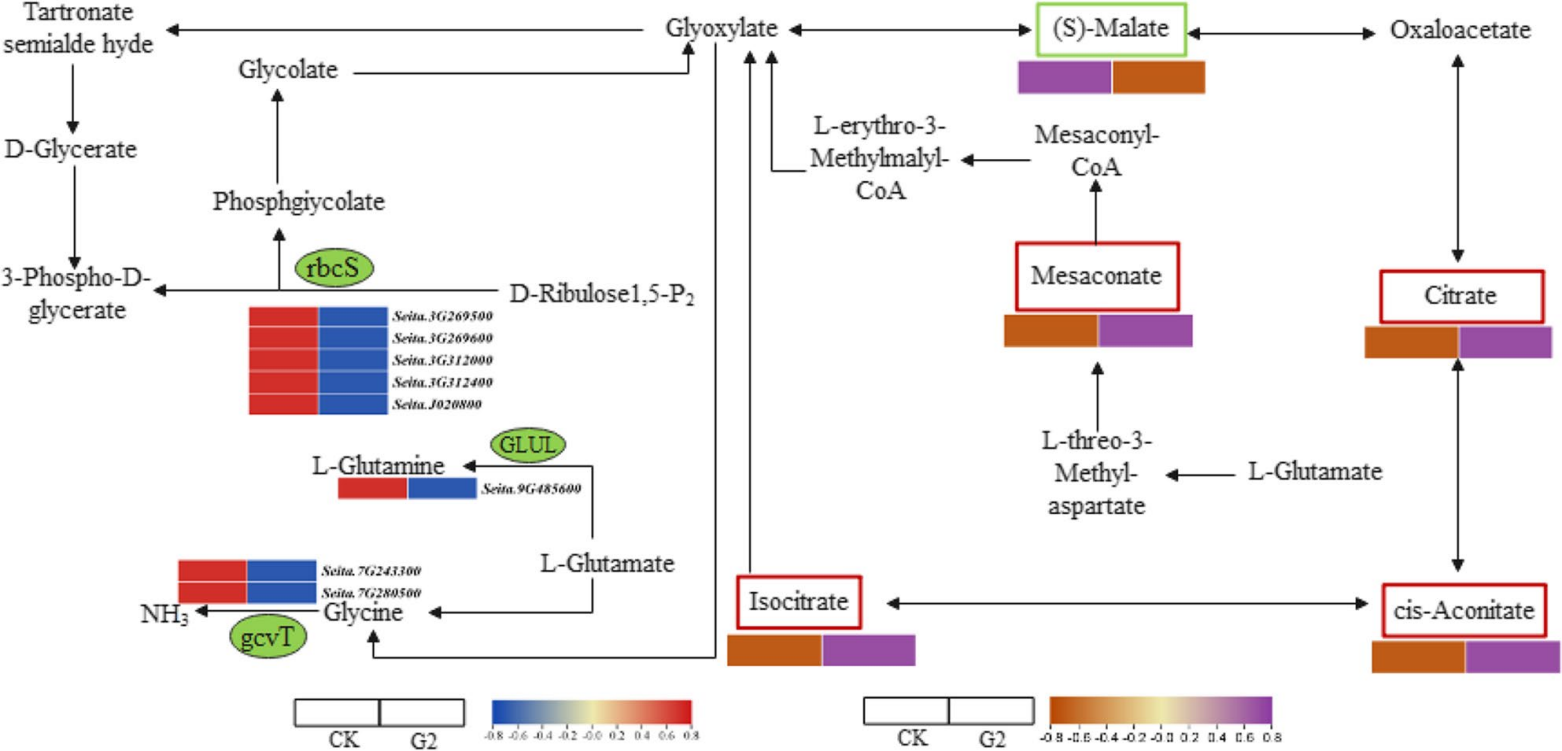
Changes in metabolism of glyoxylate and dicarboxylate pathway genes and metabolites under drought stress during the booting stage. The blue and red color blocks represent the FPKM values of DEGs, while the brownish red and purple color blocks represent changes in metabolite content

In the glyoxylate and dicarboxylate metabolism pathway, eight DEGs and five DAMs were differentially expressed or accumulated, respectively (Fig. 7). Among them, the expression levels of the *rbcS* (Seita.3G269500, Seita.3G269600, Seita.3G312000, Seita.3G312400, and Seita.J020800) which regulate the biosynthesis of 3-phospho-D-glycerate, the glutamine synthetase (*GLUL*) (Seita.9G485600) which regulates the biosynthesis of L-glutamine, and the aminomethyltransferase (*gcvT*) (Seita.7G243300 and Seita.7G280500). Genes encoding the rbcS subunit exhibited the most significant changes in expression levels. Previous studies have demonstrated that increasing the expression of *rbcS* genes can enhance plant tolerance to both biotic and abiotic stresses [33, 34]. Metabolomic results showed that the metabolites isocitrate, cis-aconitate, mesaconate, and citrate all increased to varying degrees. The metabolite (S)-malate showed a downregulation trend in their accumulation.

## Discussion

### Stage-specific sensitivity

Water is an essential component for plant growth and development, participating in vital physiological processes such as turgor maintenance, metabolic reactions, and nutrient transport [35]. Our studies exhibited that the booting stage represents a water-sensitive phase where drought stress significantly impacts yield [36], consistent with our findings demonstrating a 25.9% yield reduction under booting-stage drought. This reduction might manifest as shortened panicle length and decreased seed setting rate, primarily attributed to severe disruption of floral organ development, pollination processes, and resource allocation to reproductive structures. Similar findings have also been reported in studies on rice and other crops [37, 38]. Furthermore, drought stress drastically reduced photosynthetic production, severely limiting assimilate storage capacity while impeding endosperm cell expansion and starch accumulation-critical determinants of foxtail millet yield [39]. Previous research confirms that drought more severely inhibits photosynthesis during booting than other growth stages, with longer recovery periods required [36]. Our results suggested that the booting stage, which is critical for grain formation and development, may simultaneously modulate both grain development and leaf photosynthetic function, implying that it could be the most drought-vulnerable period in foxtail millet. Consequently, drought occurrence during this phase is likely to cause substantial declines in both yield and photosynthetic performance. Therefore, prioritizing water supply to meet booting-stage requirements might have been crucial for stabilizing foxtail millet production under water-limited conditions.

### Phenylpropanoid/lignin responses

The phenylpropanoid biosynthesis pathway may play a critical role in drought tolerance of foxtail millet. Phenylalanine ammonia-lyase (PAL) and cinnamate-CoA ligase (4CL), serving as the initiating and terminal enzymes of this pathway, coordinately drive lignin biosynthesis [40]. Significant downregulation of *CCR* inhibited cinnamaldehyde production, resulting in reduced synthesis of *p*-hydroxyphenyl lignin (H-type). This finding aligned with Ma et al., where diminished CCR activity decreased H-type lignin accumulation. *PRDX6* members exhibited differential expression (upregulation of five genes including Seita.2G431000 and downregulation of Seita.9G298500), confirming their dual roles in lignin polymerization [41, 42]. *REF1* isogenes showed antagonistic expression patterns (Seita.4G227800 upregulated, Seita.5G210300 downregulated), suggesting functional specialization and providing new perspectives for spatiotemporal precision regulation in lignin biosynthesis. Significant accumulation of sinapyl alcohol (S-lignin precursor) coupled with downregulation of phenolic compounds (*p*-coumaric acid, eugenol) aligns with the compensatory S-pathway enhancement model during H-pathway obstruction [43]. Concurrent reduction in chlorogenic acid and methyleugenol indicates drought-induced metabolic flux redirection toward lignin biosynthesis. Our study suggested a potential *REF1* isogene specialization mechanism, providing preliminary insights into a possible plant adaptation strategy under stress conditions — where chemical defense (via phenolics) may be compromised to reinforce structural defense (lignin biosynthesis).

Photosynthesis serves as the core process for dry matter accumulation and yield formation. Drought stress suppresses its function through dual pathways: stomatal limitation (reduced stomatal) conductance and non-stomatal limitation (photosystem damage) [13, 29, 44, 45]. This study revealed particularly significant expression changes in photosynthetic carbon fixation-related genes under drought stress. The Calvin cycle, as the central pathway for photosynthetic carbon assimilation, fixes and transforms carbon sources through three coordinated phases: carboxylation, reduction, and regeneration. Powered by light reactions, it ultimately generates 3-phosphoglyceraldehyde of a key precursor for carbohydrate synthesis [46]. GAPA consumes ATP and NADPH to catalyze the conversion of 3-phosphoglycerate to glyceraldehyde-3-phosphate, providing essential carbon skeletons for metabolism [47]. The chloroplast-localized subunit encoded by the *GAPA* gene acts as a key regulator in response to oxidative stress and abscisic acid signaling [48, 49]. *ALDO* functions as a rate-limiting enzyme constraining photosynthetic rate and carbon flux [50, 51]. In this study, *tkta* was in an upward trend, while *GAPA*, *ALDO*, *glpx*, *RPE*, *PRK*, *rbcS*, and *maeB* were simultaneously decreased. The results suggested that under drought stress at the booting stage, the supply of 1,3-diphosphoglyceric acid may be inhibited during glycolysis in foxtail millet, which could prevent the carbon fixation process from functioning normally. Additionally, foxtail millet exhibited an adaptive strategy featuring photosynthetic suppression coupled with enhanced carbon translocation. This provided valuable insights for future research on drought-resistant gene functions.

### C–N regulation and glyoxylate/TCA cycle remodeling

Our study provided preliminary insights into a potential core mechanism underlying the imbalance of carbon-nitrogen co-regulation in the glyoxylate-dicarboxylate metabolic pathway under drought stress. Carbon fixation limitation is primarily manifested in the downregulation of the *rbcS* gene family leading to reduced synthesis of 3-phospho-D-glycerate (3-PGA) and decreased carbon fixation efficiency in the Calvin cycle. This finding aligned with previous research, as Nascimento et al. [52] confirmed that *rbcS* suppression reduces photosynthetic carbon flux, forcing cells to mobilize stored carbon sources. The downregulation of the *GLUL* and *gcvT* may impair N assimilation capacity, restricting L-glutamine and glycine metabolism. Simultaneously, the co-downregulation of *GLUL* and *gcvT* dual-blockades N assimilation — limiting glutamine synthesis [53] — while *gcvT* downregulation impairs the glycine cleavage system and reduces NH₃ regeneration [54]. This aligns with Peng et al. [55], who reported that N-deficient plants redistribute resources by suppressing core carbon-nitrogen genes. Metabolomic changes further corroborated these mechanisms. The accumulation of TCA cycle intermediated (isocitrate, cis-aconitate, citrate) reflects mitochondrial metabolic remodeling due to reduced carbon input slowing cycle turnover [56]. Meanwhile, the downregulation of malate-a critical bridge linking the TCA and glyoxylate cycles-weakens oxaloacetate replenishment, exacerbating energy crisis [57]. Additionally, the abnormal accumulation of mesaconate was a striking finding of this study. This metabolite is rare in plants but abundant in the microbial methylmalonate pathway [58], suggesting the potential activation of novel stress-response pathways in foxtail millet. This will be a key focus of our follow-up research.

### Limitations and future directions

This study has several limitations that need to be explicitly acknowledged. First, only a single foxtail millet genotype (*Jigu 45*) was used in the experiment, which may limit the generalizability of the findings to other foxtail millet varieties with distinct genetic backgrounds. Second, although a large number of DAMs were identified through metabolomic profiling, only a limited number of these metabolites were chemically validated, which may affect the reliability of some metabolic annotation results. Third, despite the observation of nitrogen metabolism-related changes (e.g., downregulation of *gcvT* and *GLUL* genes), N metabolites were not comprehensively quantified, preventing a thorough understanding of the N metabolic remodeling process under drought stress. Fourth, the microbiome, which plays an important role in plant stress response and nutrient acquisition, was not assessed in this study, thus missing the potential interactions between the plant and its associated microorganisms during drought adaptation. These limitations provide directions for future research: expanding the study to multiple genotypes, conducting targeted chemical validation of key metabolites, performing comprehensive quantification of N-related metabolites, and integrating microbiome analysis to gain a more holistic insight into the drought response mechanisms of foxtail millet at the booting stage.

## Conclusions

To decipher the molecular mechanisms underlying drought response during the booting stage in foxtail millet, this study measured photosynthetic and yield-related parameters. We identified distinct drought response patterns across growth stages, with drought stress at the booting stage exerting the most pronounced negative impact on yield. Integrated transcriptomic and metabolomic analyses revealed three key pathways modulated by booting-stage drought: Gene *PRDX6*, *CCR*, and *REF1* regulated key metabolites *p*-hydroxy-phenyl lignin, eugenol, guaiacyl lignin, and sinapyl alcohol within phenylpropanoid biosynthesis pathway. Gene *maeB* mediated regulation of metabolites aspartate and malic acid, combined with *tkta*, *GAPA*, *ALDO*, *glpx*, *PRK*, and *rbcS* driven modulation of sedoheptulose, collectively modulates the carbon fixation pathway in photosynthetic organisms. Genes *rbcS*, *GLUL*, and *gcvT* coordinated with metabolites isocitrate, cis-aconitate, mesaconate, citrate to regulate glyoxylate synthesis and degradation. These findings provided critical mechanistic insights into the molecular basis of drought tolerance in foxtail millet, identifying strategic targets for enhancing crop resilience in arid environments.

## Materials and methods

### Plant materials and experimental design

The foxtail millet cultivar *Jigu 45*, cultivated by the Millet Research Institute of Hebei Academy of Agriculture and Forestry Sciences, was used in this study. Trials were conducted in rainout shelters at *Mazhuang* Experimental Station (37°93’N, 114°78’E) in 2024. The physicochemical properties of the topsoil (0 – 20 cm plough layer) at sowing were as follows: total N 16.58 g/kg, available N 104.62 mg/kg, organic matter 20.36 g/kg, available potassium (K) 98 mg/kg, available phosphorus (P) 22.91 mg/kg, and pH 7.91. The soil parent material was deep Quaternary sediments with a thickness of more than 700 to 1000 m. According to the World Reference Base for Soil Resources (WRB) classification system, the soil was classified as calcareous alluvial soil. The soil texture was sandy loam, with sand, silt and clay accounting for 63.67%, 18.83%, and 17.5% of the particle composition, respectively.

Four treatments, including normal irrigation (CK), drought at jointing stage (G1), drought at booting stage (G2), and drought at filling stage (G3), were set up, with a length of 3 m, a row spacing of 40 cm, six rows per plot, a randomized block design, three individual biological replicates (corresponding to three independent blocks), and a total of 12 plots. The planting density was 40,000 seedlings per mu. Basal application included 112.26 kg organic fertilizer and 16.84 kg compound fertilizer per mu, with all other field management practices following conventional protocols. For soil water content control, the soil water content of all drought treatment groups (G1, G2, G3) was reduced to 35% ± 5% of field capacity, while that of the control group (CK) was maintained at 60% – 70% of field capacity throughout the entire growth period via regular irrigation; specifically, irrigation was stopped when plants entered the jointing stage, booting stage, and filling stage for G1, G2, and G3, respectively, and drought stress was maintained until the end of the corresponding growth stage. For the drought treatment groups, during non-treatment periods (i.e., before the start of targeted drought stress and after the end of the drought period), the soil water content was consistent with CK (60% – 70% field capacity) to ensure uniform growth conditions across all groups except during the specified drought stress periods.

### Determination of photosynthetic and yield indices at different growth stages

Photosynthetic indices were measured on the seven days after drought stress treatment, with the functional flag leaves of plants selected as the measurement objects. The relative SPAD value was determined using a portable chlorophyll meter (SPAD-502PLUS, Konica Minolta, Japan). The net photosynthetic rate (Pn), conductance to H_2_O (Gs), and transpiration rate (Tr) were measured using the LI-6400XT portable photosynthesis system (Li-COR, distributed by Beijing Ligota Technology Co., Ltd.). All measurements were performed with three biological replicates (corresponding to three independent blocks, one representative plant selected per block).

Yield-related indices were determined after maturation. Three representative plants were selected from each plot, and their spikes were harvested to measure spike length, spike diameter, spike weight, and grain weight per spike. Meanwhile, a continuous four-row area with a row length of 2 m was selected from each plot for yield measurement. Based on the yield measurement results, the yield per unit area was calculated by conversion.

### RNA extraction and transcriptome sequencing

Flag leaves of foxtail millet plants subjected to CK and G2 treatments were collected for transcriptome analysis. Each sample was derived from one representative plant per independent experimental block, with three biological replicates (three blocks) set for each treatment. RNA extraction was performed following the instructions of the TaKaRa RNAiso Plus Total RNA Extraction Kit, and subsequent sequencing was conducted on the Illumina HiSeq 2000 platform, with a read length of 150 base pairs (bp) (Illumina, USA). Fastp (https://github.com/OpenGene/fastp), HISAT2 (http://ccb.jhu.edu/software/hisat2/index.shtml), and RSEM (http://deweylab.github.io/RSEM/) were employed for subsequent quality control, alignment to the reference genome, and expression analysis. DEGs were identified by performing pairwise comparisons between the sample treatments (DESeq2, Fold Change ≥ 2, and FDR < 0.05. Subsequently, GO functional enrichment analysis and KEGG pathway enrichment analysis were performed on the identified DEGs. This allowed for the acquisition of protein functional annotations and functional classification statistics corresponding to the DEGs on Majorbio platform [59].

### Metabolome analysis

Flag leaves of foxtail millet plants from each treatment group were selected for broad-targeted metabolomic analysis, with each sample comprising six biological replicates (three independent experimental blocks, two representative plants per block). Liquid chromatography–tandem mass spectrometry (LC-MS/MS) analysis was performed. The LC-MS analysis in this study was conducted using an ultra-high-performance liquid chromatography (UPLC) system coupled to a Fourier transform mass spectrometer (FTMS) from Thermo Fisher Scientific (Thermo, USA). Following LC-MS analysis, the raw data were imported into the metabolomics data processing software Progenesis QI (Waters Corporation, Milford, USA) for processing. This included baseline filtering, peak picking, peak integration, retention time (RT) correction, and peak alignment. Ultimately, a three-dimensional data matrix comprising RT, mass-to-charge ratio (m/z), and peak intensity was generated. Concurrently, the acquired MS and MS/MS spectra were matched against public metabolite databases-specifically, the HMDB (http://www.hmdb.ca/) and Metlin (https://metlin.scripps.edu/)-as well as the in-house metabolite database, thus obtaining the corresponding metabolite annotations. The variable importance in projection (VIP) parameter was used to evaluate the relative importance of each metabolite to the PLS-DA model. Metabolites with VIP ≥ 1 and |log_2_FC| ≥ 1 were considered differential metabolites for group discrimination [59]. This analysis was commissioned to Shanghai Majorbio Bio-Pharm Technology Co. Ltd.

### Data analysis

Excel 2016 and Origin 2021 were used for data calculation and graphing, and DPS 7.05 software was applied for significance analysis.

## Supplementary Information

Supplementary Material 1.

## Data Availability

The datasets presented in this study can be found in online repositories. The names of the repository/repositories and accession number(s) can be found below: https://www.ncbi.nlm.nih.gov/sra/PRJNA1335833.

## Abbreviations

CK: Control check
DAMs: Differentially accumulated metabolites
DEGs: Differentially expressed genes
FDR: False discovery rate
FPKM: Fragments per kilobase of transcript per million mapped reads
G1: Drought at jointing stage
G2: Drought at booting stage
G3: Drought at filling stage
GO: Gene Ontology
KEGG: Kyoto Encyclopedia of Genes and Genomes
Pn: Net photosynthetic rate
ROS: Reactive oxygen species
VIP: Variable importance in projection
WUE: Water-use efficiency
WRB: World Reference Base for Soil Resources
